# Physical activity and anodal-transcranial direct current stimulation: a synergistic approach to boost motor cortex plasticity

**DOI:** 10.1093/braincomms/fcaf167

**Published:** 2025-05-06

**Authors:** Federica Marchiotto, Marco Cambiaghi, Mario Buffelli

**Affiliations:** Department of Neurosciences, Biomedicine and Movement Sciences, University of Verona, Verona 37134, Italy; Department of Neurosciences, Biomedicine and Movement Sciences, University of Verona, Verona 37134, Italy; Department of Neurology, Hackensack Meridian School of Medicine, Nutley, NJ 07110, USA; Department of Neurosciences, Biomedicine and Movement Sciences, University of Verona, Verona 37134, Italy

**Keywords:** primary motor cortex, anodal tDCS, C57BL/6 mice, dendritic spine density, local field potential coherence

## Abstract

The application of anodal-transcranial direct current stimulation (A-tDCS) over the primary motor cortex (M1) increases its structural and functional plasticity, as also physical exercise. Combining both interventions has a boosting effect, thus revealing a crucial role of the brain state during stimulation. Although brain slice and anesthetized animal studies support this, further investigation in awake animals is necessary. In the present study, we analyzed the effects of coupling A-tDCS with low-intensity physical activity on the mouse M1 structural and functional plasticity. C57BL/6 mice were monolaterally treated with M1 A-tDCS while walking on a rotarod or at rest. To assess the impact of our interventions, we analyzed both motor cortices for changes in neuronal activation, dendritic spine density, and functional synchronisation as measured by local field potential coherence. The combination of physical activity and M1 stimulation revealed a synergistic interhemispheric effect on cortical activation in both layers II/III and V, not present when using a single type of intervention. These data were accompanied by increased M1-M1 synchrony in the low-theta frequency, a hallmark of motor network activity in mice. Dendritic spine density revealed an effect of the combo, which was significantly higher only in layer II/III, accompanied by increased post-synaptic density protein 95 expression in the same area. Based on our findings, we propose that the efficacy of tDCS hinges on brain state rather than being merely a direct causal factor. The observed outcomes contribute to a deeper comprehension of the mechanisms governing structural and functional reorganisation within the motor cortex under physiological conditions, with potential implications for research on learning, memory, and neurological disorders such as stroke.

## Teaser

Marchiotto *et al*. revealed the indirect effects of anodal-transcranial direct current stimulation on the mouse motor cortex, when simultaneously associated with low-intensity physical activity. Combining walking and stimulation significantly boosts motor cortex plasticity. These findings suggest that the brain's state is crucial for transcranial direct current stimulation effectiveness.

## Graphical Abstract

**Graphical Abstract:**
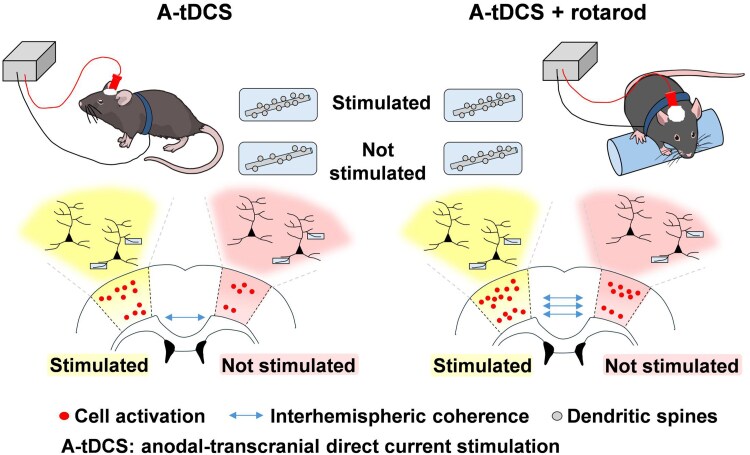

## Introduction

The brain state refers to the dynamic pattern of neural activity and connectivity at a given moment, determined and influenced by factors such as cognitive processes, sensory inputs, and ongoing brain oscillations.^1^ Over the last decade, an increasing awareness has emerged of the importance of the brain state during the application of non-invasive neuromodulatory techniques, such as transcranial direct current stimulation (tDCS), in determining their aftereffect.^2-4^ Early evidence from motor cortex studies showed that anodal and cathodal tDCS had opposite effects, the former increasing and the latter decreasing primary motor cortex (M1) excitability, outlasting the stimulation period in both humans^5^ and mice.^6^ Nevertheless, in the mouse M1 slice model, anodal stimulation induced long-lasting synaptic potentiation exclusively when combined with concurrent, repeated weak synaptic activation (layer V → II/III electrical stimulation at 0.1 Hz).^7^ Similarly, in the sensorimotor cortex, anodal tDCS (A-tDCS) effectively enhanced dendritic spine structural plasticity when combined with the contralateral forepaw electrical stimulation (0.5 mA at 0.1 Hz), in anesthetized mice.^8^ Therefore, to successfully modulate M1 in both ex vivo slices and anesthetized mice, tDCS needs the concomitant application of synaptic inputs (i.e. electrical stimulation), strongly suggesting the importance of the brain state during stimulation. In behaving mice at rest, the application of A-tDCS results in increased dendritic spine density of layer II/III pyramidal neurons, enhanced long-term potentiation, and better performance in a motor task.^9^ Indeed, in the awake animal motor cortical activity is correlated with the level of behavioural arousal and/or the concurrent motor task.^10^ Acquired motor tasks correlate with morphological modifications in the motor areas, with the formation and conservation of dendritic spines being associated with the degree of acquisition and maintenance of the motor skill, assumed to provide a new physical basis for storing motor memories.^11,12^ Within M1, changes in spine density underlie circuits rewiring leading to synaptic plasticity, thereby influencing synaptic transmission efficacy and long-term changes.^13^ Therefore, an increase in M1 dendritic spine density represents the structural synaptic changes associated with functional synaptic strengthening, known to be linked to motor/behavioural learning.^12,14^ A large number of human studies combining A-tDCS with physical training showed improved M1 excitability, physical performance, and motor learning if compared with each single action, revealing a synergistic effect of the two components.^15^ While these results provide valuable insights, further investigation through *in vivo* experiments on behaving animals is needed to elucidate the influence of endogenous motor cortex activity on direct stimulation outcomes.

Moreover, while tDCS primarily modulates the designated target region beneath the electrode, it indirectly affects interconnected brain regions, inducing a transient reconfiguration of the underlying neural network. Research utilising fMRI in human M1 has indicated that unilateral tDCS can modulate brain activity in remote areas, such as the contralateral motor cortex,^16,17^ though not producing substantial alterations in evoked motor potentials, consistent with findings from previous studies.^18^ Additionally, ex vivo studies have shown that DCS applied concurrently with synaptic activity promotes cumulative neuromodulation, thereby enhancing synaptic cooperativity.^3^ Recently, in the anesthetized mouse, we found an indirect and immediate effect of prefrontal tDCS on a distant interconnected area such as the dorsal raphe nucleus.^19^

Taken together, these various aspects are of particular interest not only for a better understanding of tDCS neurobiology and its influence on motor network activity but also in the perspective of optimal use of this tool in rehabilitative medicine, such as after a stroke or traumatic brain injury. Therefore, the main aim of the present study was to investigate the effects of monolateral A-tDCS on the activated versus resting motor cortex, analyzing its functional interaction and the structural plasticity of dendritic spines.

## Materials and methods

### Animals and experimental groups

A total of 64 male C57BL6 mice aged 10–12 weeks were used for this study. The animals were group housed under a 12:12 h light/dark cycle at a controlled temperature, with free access to food and water. Mice were randomly assigned to one of the following experimental groups, according to the stimulation protocol (sham versus A-tDCS) and physical activity (home cage versus rotarod): (i) Sham/r−, which received sham stimulation in their home-cage; (ii) Sham/r+, sham-treated mice performing rotarod, (iii) A-tDCS/r−, A-tDCS mice while in their home-cage; (iv) A-tDCS/r+, stimulated mice while walking on the rotarod. After surgery, mice were single-housed for the rest of the experiment. All animal procedures were performed in accordance with the EU Council Directive 2010/63/EU on the protection of animals used for scientific purposes and were approved by the Italian Ministry of Health, according to the ARRIVE guidelines.

### Electrode implantation and tDCS protocol

**Figure 1:**
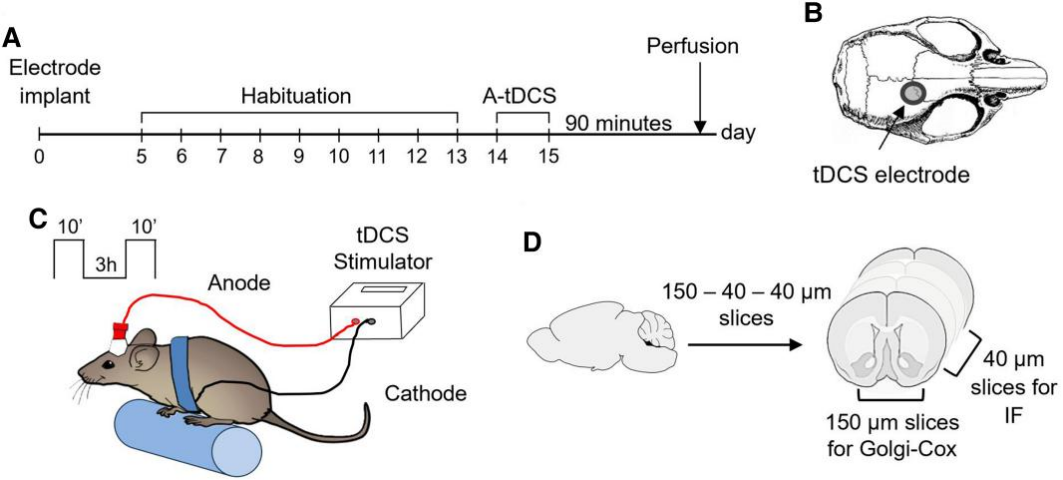
**Experimental design and stimulation procedure.** (**A**) Plan of the experimental timeline. After electrodes implant, mice underwent a 9-day habituation before tDCS. Ninety minutes after the last stimulation, mice were perfused, and the brains were collected. (**B**) Schematic image of tDCS active electrode location. (**C**) Representative image of A-tDCS/r+ condition, where the mouse walks on the rotarod while stimulated for 10 min, two times with 3 h break, for two consecutive days. (**D**) After the dissection, brains were sliced according to a 150µm-40µm-40µm pattern to perform immunofluorescence staining and Golgi-Cox technique on the same sample.

Mice were anesthetized by an intraperitoneal injection of ketamine-xylazine (80 mg/kg and 5 mg/kg, respectively) and placed on the stereotaxic frame. A tubular plastic jack (inner area 4.5 mm^2^) was dental cemented over the right M1 area, 1 mm anterior and 2 mm lateral to Bregma.^6^ After surgery, all animals were allowed to recover for 3–4 days. Before entering the stimulation procedure, all the mice started a 9-day habituation to the rotarod apparatus and were then randomly divided into groups. To deliver A-tDCS the electrode was filled with saline solution (0.9% NaCl), while the counter electrode consisted of a saline-soaked sponge applied over the ventral thorax using a rubber-made corset. Stimulation was applied at a current intensity of 250μA for 10 min twice a day (3 h break) for two consecutive days (Fig. 1A–D). The current intensity was ramped instead of switching it on and off immediately to avoid a stimulation break effect. Sham groups of animals underwent the same protocol, but no current was delivered. Sham/r− and A-tDCS/r− mice remained in their home cage, while Sham/r+ and A-tDCS/r+ underwent sham or A-tDCS with the rotarod speed maintained constant at 9 rpm (1.8 cm/s).

### Histological procedures

Ninety minutes after the last stimulation, mice were transcardially perfused with 4% paraformaldehyde. Collected brains were post-fixed and stored. Serial coronal sections containing the M1 were cut using a vibratome (Leica VT1200) with a repetitive thickness pattern of 150μm-40μm-40μm (Fig. 1D). For each animal, three 40μm sections within M1 (+1.94, +1.50, +1.10 from Bregma) were processed for immunofluorescence staining to evaluate cell activation with the early-gene marker cFos (1:200, Synaptic Systems anti-rabbit, cat. 226003) and the post-synaptic density with the scaffolding protein PSD95 (1:400, GeneTex anti-rabbit, cat. GTX133091) primary antibody, followed by an anti-rabbit 594 (1:1000, Sigma cat. SAB4600107) and an anti-rabbit 647 (1:1000, Sigma cat. SAB4600184), respectively.

### Analysis of immunolabeled neurons

To measure cell activation, images of layer II/III and layer V of the left and right M1 were acquired by using Leica Fluorescence Microscope (Leica Systems, Wetzlar, Germany) with a 20X objective lens (ROIs of 1392 × 1040 pixels size). cFos+ cells were manually counted by an operator unaware of the experimental group. To analyze the PSD95 signal, two *z*-stacks images (1024 × 1024 pixels, step size 0.5 μm, *z*-stack thickness 5 μm) of layer II/III and layer V of left and right M1 were acquired with Leica-Sp5 Confocal microscope with a 63X glycerol immersion objective at 1.5 zoom. Later, two ROIs from each image were randomly selected (288 × 266 pixels size), and the percentage of area fraction occupied by the PSD95 signal was calculated with ImageJ (Fiji).

### Golgi staining and dendritic spine analysis

For the dendritic spine density analysis, 150 μm slices were processed for Golgi-Cox staining. Two M1 sections/animal were immersed in Golgi-Cox solution, made by mixing Solution A and B of Rapid Golgi-Cox Staining Kit. After 14 days, slices were treated firstly with Ilfosol3 (Ilfosol, cat. 1131778, Ilford; 1 Ilfosol + 9 H2O parts) and then with Rapid Fixer (Ilford, cat. 1984253; 1 Rapid Fixer + 9H2O parts). After dehydration, slices were mounted with Eukitt Quick-hardening mounting medium (Sigma-Aldrich, cat. 25608-33-7). Dendritic spines of both right and left M1 cortical pyramidal neurons were manually counted using a Leica Fluorescence Microscope (Leica Systems, Wetzlar, Germany) with a 100X oil-immersed objective lens in brightfield. In layer II/III we analyzed the distal segments of tertiary apical and secondary basal dendrites. In layer V, we investigated secondary basal dendrites and tertiary apical dendrites extending in layers II/III and V.^20^ Dendritic spine density was calculated by dividing the number of spines over the dendritic length in µm.

### *In vivo* local field potential recordings and analysis

Extracellular field potentials were recorded in awake mice while resting, at baseline (Day 8, pre-stimulation) and after the stimulation protocol (Day 15, post-stimulation). Local field potential (LFP) was recorded with tungsten wires (ø 50 μm) implanted in the right and left M1 cortex, according to the following coordinates: AP = + 1.1 mm, ML = ±1.6 mm from Bregma and DV = −0.5 mm from the brain surface, with the reference electrode over the cerebellum. Cresyl Violet staining was performed to verify the location of the electrodes.

A customized Python script was used for offline coherence analysis, for which three 2-second epochs were averaged. As in our previous paper,^21^ differences in coherence were obtained by subtracting mean coherence values (post-stimulation—pre-stimulation) and measured within the following frequency bands: delta (0.5–3.5 Hz), theta-1 (4–8 Hz), theta-2 (8.5–12 Hz), alpha (12.5–15 Hz) and gamma (15.5–20 Hz).

### Cholera toxin subunit B injection, histology, and analysis

To label M1 neurons projecting to the contralateral motor cortex, 0.2 µL of Cholera Toxin Subunit B (CTB) Alexa Fluor 647 Conjugate (cat. C34778, Thermo Fisher Scientific) was injected in the right M1 (AP = + 1.1 mm, ML = + 1.6 mm, DV = −0.6 mm from Bregma), by using a micro-capillary tube. After 3 days, mice were transcardially perfused, brains collected, and 40 µm coronal sections cut and mounted. Each M1 section was imaged at 10× using a fluorescent microscope (Olympus APX100). Multi-images at 10X allowed the reconstruction of the entire section.

### Statistical analysis

Adequate sample size was determined according to our previous studies with analogous experiments.^19,22^ Statistical analysis was performed using Prism 8.3 Software (GraphPad, La Jolla, CA, USA). After normality distribution testing (Shapiro-Wilk test), inter-hemispheric and inter-groups data were analyzed with a two-tailed unpaired *t*-test or with ordinary one-way ANOVA with Tukey’s multiple comparisons test, unless differently indicated. Results are presented as mean ± SEM, and differences among groups were considered statistically significant with a *P* < 0.05.

## Results

In the sliced mouse motor cortex, A-DCS coupled with low-frequency synaptic activation promotes long-lasting synaptic plasticity, which is not observed when applying DCS alone.^7^ Similarly, in the anesthetized mice, A-tDCS results in enhanced structural synaptic plasticity only if simultaneously coupled with motor cortex activation (i.e. 0.1 Hz contralateral forepaw electrical stimulation).^8^ In healthy volunteers, motor performance is improved when tDCS is combined with physical training.^15^ Accordingly, tDCS effects are not considered one-size-fits-all, but the concurrent neural activity within the motor cortex is found to play a key role in its response to stimulation.^1^ Moreover, locomotion and other motor behaviours rely on intercortical connections that bridge the brain hemispheres via the corpus callosum, mutually affecting each other's activity.^23^ Here, we addressed whether monolateral A-tDCS during walking could modulate activity in the unstimulated contralateral motor cortex. To this aim, we applied A-tDCS over the right mouse M1 during low-intensity locomotion or home-cage resting for two consecutive days, and we examined both hemisphere activation, functional interaction, and dendritic spine modulation (Fig. 1).

### Coupling A-tDCS and physical activity enhances motor cortex activation and functional interaction

**Figure 2:**
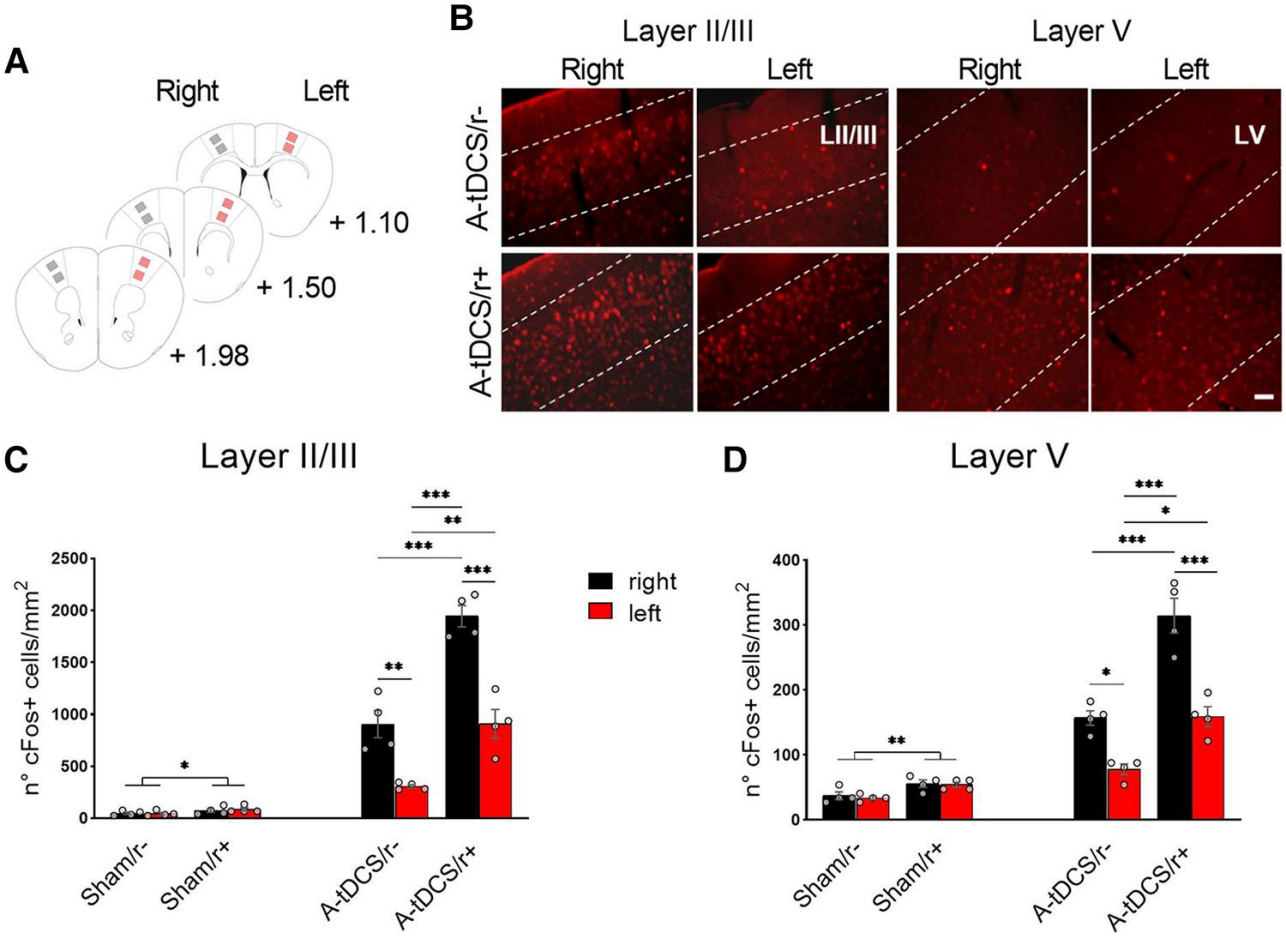
**Combining monolateral A-tDCS and walking increases M1 cortices activity.** (**A**) Schematic of regions of interest used for cFos+ cells count (stimulated side in grey; non-stimulated side in red). Images of layers II/III and V were acquired in three M1 sections (+1.98; +1.50; +1.10 mm from Bregma) in both the right and left hemispheres. (**B**) Representative layer II/III and layer V M1 cFos staining. Images were acquired at 20X objective with a fluorescent microscope. Scale bar = 20 µm. (**C, D**) The number of cFos + cells in M1 increased after stimulation in both hemispheres when coupled with endogenous activation of the motor cortex by walking (*N* = 4/group; grey dots indicate individual values), in layer II/III (**C**) Two-way ANOVA followed by Tukey’s *post hoc* correction. Treatment × hemisphere interaction: *F*_3,24_ = 21.25, *P* < 0.0001; treatment factor: *F*_3,24_ = 137.9, *P* < 0.0001; hemisphere factor: *F*_1,24_ = 54.64, *P* < 0.0001 and in layer V (**D**), Two-way ANOVA followed by Tukey’s *post hoc* correction. Treatment × hemisphere interaction: *F*_3,24_ = 17.43, *P* < 0.0001; treatment factor: *F*_3,24_ = 106.8, *P* < 0.0001; hemisphere factor: *F*_1,24_ = 46.75, *P* < 0.0001. Walking without stimulation results in a moderate increase in cell activation. Data are expressed as mean ± SEM. * *P* < 0.05; ** *P* < 0.01; *** *P* < 0.001.

**Figure 3:**
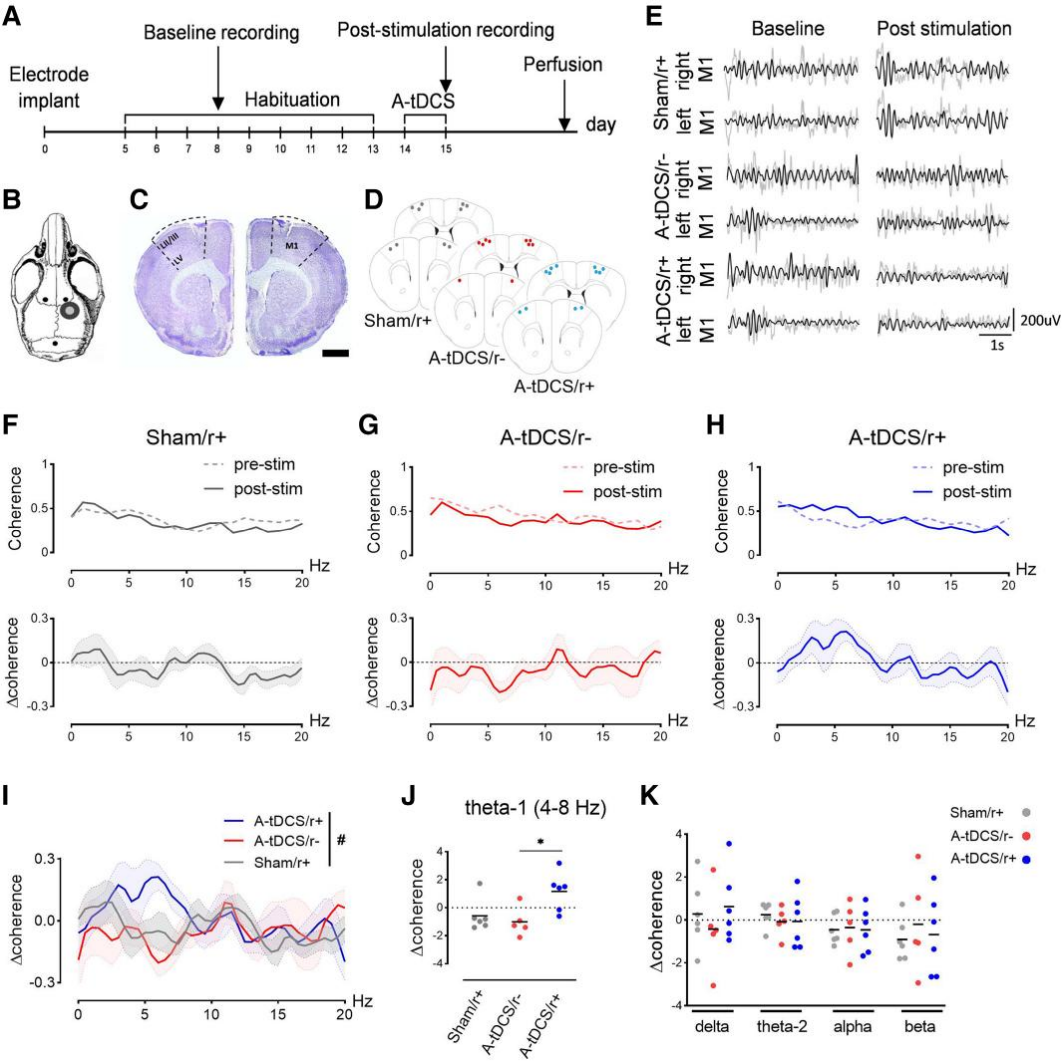
**M1-M1 increased synchrony after A-tDCS combined with physical activity.** (**A**) Experimental timeline indicating the protocol used for LFP recordings. The baseline recording was done on Day 8 (pre-stimulation), and the post-stimulation recording on Day 15, after the last stimulation. (**B**) Schematic brain image depicting the location of the implanted M1 electrodes and the cerebellar reference electrode (in black). The plastic tubular jack for tDCS is placed in grey. (**C**) Representative histology of M1 electrode tracks. Scale bar = 1 mm. (**D**) Localisation of electrodes’ tips in sections +1.50 and +1.10 mm from Bregma. (**E**) Representative LFP traces of baseline and post-stimulation recordings (grey) in the right/left M1 in Sham/r+, A-tDCS/r−, and A-tDCS/r+ with superimposed 4–8 Hz filtered trace (black). (**F–H**) The difference in M1-M1 coherence between post-stimulation and baseline (Δcoherence) was increased only in A-tDCS/r+ (**H**), if compared with Sham/r− (**F**; *N* = 6) and A-tDCS/r− (**G**; *N* = 5), with a peak at 6 Hz (**I**¸ N = 6) (Two-way ANOVA with Sidak *post hoc* correction. Interaction: *F*_80,574_ = 0.7711, *P* = 0.926; Groups: *F*_2574_ = 4.010 *P* = 0.018; frequencies: *F*_40,574_ = 0.7751, *P* = 0.8396). Insets represent the absolute coherence of baseline (dotted line) and post-stimulation (filled line); black lines indicate the coherence expected by chance. (**J**) Low-theta (4–8 Hz) coherence is significantly higher only when tDCS and physical activity are combined, but not in the other frequency ranges (**K**) Each dot indicates a single animal; black lines indicate the median value. Data are expressed as mean ± SEM. * *P* < 0.05.

The application of tDCS activates the expression of cFos both beneath the electrode and in interconnected regions.^19^ We thus asked whether monolateral A-tDCS affects the contralateral M1 activity when combined with the physiological activation of the entire motor cortex (i.e. walking). To answer this question, in the stimulated (right) and non-stimulated (left) M1 (Fig. 2A), we tested cFos levels (as a measure of cell activation) in layers II/III and V while mice were walking or at rest (Fig. 2B), with or without concurrent A-tDCS (Supplementary Tables 1 and 2). Since motor activity is known to induce cFos expression in M1,^24^ we first examined the effects of walking by comparing non-stimulated mice in the home-cage (Sham/r−) with the ones walking on the rotarod (Sham/r+). In both layers II/III and V, physical activity increased the number of cFos + cells (*P* = 0.04 and *P* = 0.001, respectively; Fig. 2 and Supplementary Fig. 1). When applied in humans and animals, monolateral M1 A-tDCS enhances neural activity mainly in the stimulated hemisphere, as evidenced by MEPs or IEGs.^5,6,25^ Consistent with these findings, we observed that monolateral M1 stimulation in resting mice (A-tDCS/r−) had increased cell activation of the ipsilateral (i.e. right) with respect to the contralateral M1 in layer II/III and V (*P* = 0.0003 and *P* = 0.003, respectively; Fig. 2C, D). Then, we considered the synergistic effects of A-tDCS and motor activity (A-tDCS/r+), a procedure known to improve motor cortex functionality compared with tDCS-or-exercise/training-only intervention in humans.^15^ In this group, we observed higher cell activation in the stimulated hemisphere when compared with A-tDCS/r− in both layers II/III (*P* < 0.0001; Fig. 2C) and V (*P* < 0.0001; Fig. 2D), demonstrating that the brain state strongly contributes to tDCS outcomes. Interestingly, while remaining a significant interhemispheric difference in A-tDCS/r+ in both layers (*P* < 0.0001), the non-stimulated M1 in the A-tDCS/r+ showed a higher activation with respect to the non-stimulated M1 in the A-tDCS/r− in both layers (*P* = 0.0003 and *P* = 0.002, respectively; Fig. 2C, D), suggesting an indirect enhancing effect of A-tDCS. This outcome prompted us to explore whether the bilateral M1 enhancement might be associated with interhemispheric functional changes. To this aim, we investigated M1-M1 connectivity by measuring LFP coherence as an index of interhemispheric synchronous activity.^26^ While pyramidal neurons in the upper layers, i.e. layer II/III, mostly project to the contralateral cortex layers II/III and V, layer V neurons also project outside the neocortex to reach subcortical targets, forming the corpus callosum.^27^ Retrograde tracing with CTB-A647 from the right M1, aligned with LFP recording sites, was adopted to confirm monosynaptic projections at our coordinates, which were observed in both contralateral cortical layers II/III and V (Supplementary Fig. 2). Baseline M1-M1 coherence was recorded immediately after the first day of walking on the rotarod (Day 8) and compared with the post-stimulation recording, executed immediately after the last A-tDCS session (Day 15) (Fig. 3A–E). For each condition, post-stimulation—baseline M1-M1 synchrony (Δ coherence) was compared among groups for each frequency band (Fig. 3F–H). This analysis did not report any difference between Sham/r+ and A-tDCS/r− mice for each frequency band, while A-tDCS/r+ mice revealed increased coherence levels in the low-theta band (4–8 Hz, Kruskal-Wallis test, *P* = 0.02), but not in the other frequency ranges (Fig. 3I–K). Our findings indicate that combining A-tDCS with physical activity enhances synchronisation within the motor cortex at the theta frequency, a hallmark of motor network connectivity.^28^

### A-tDCS combined with a physiological motor cortex activation boosts dendritic spine density in both hemispheres

**Figure 4:**
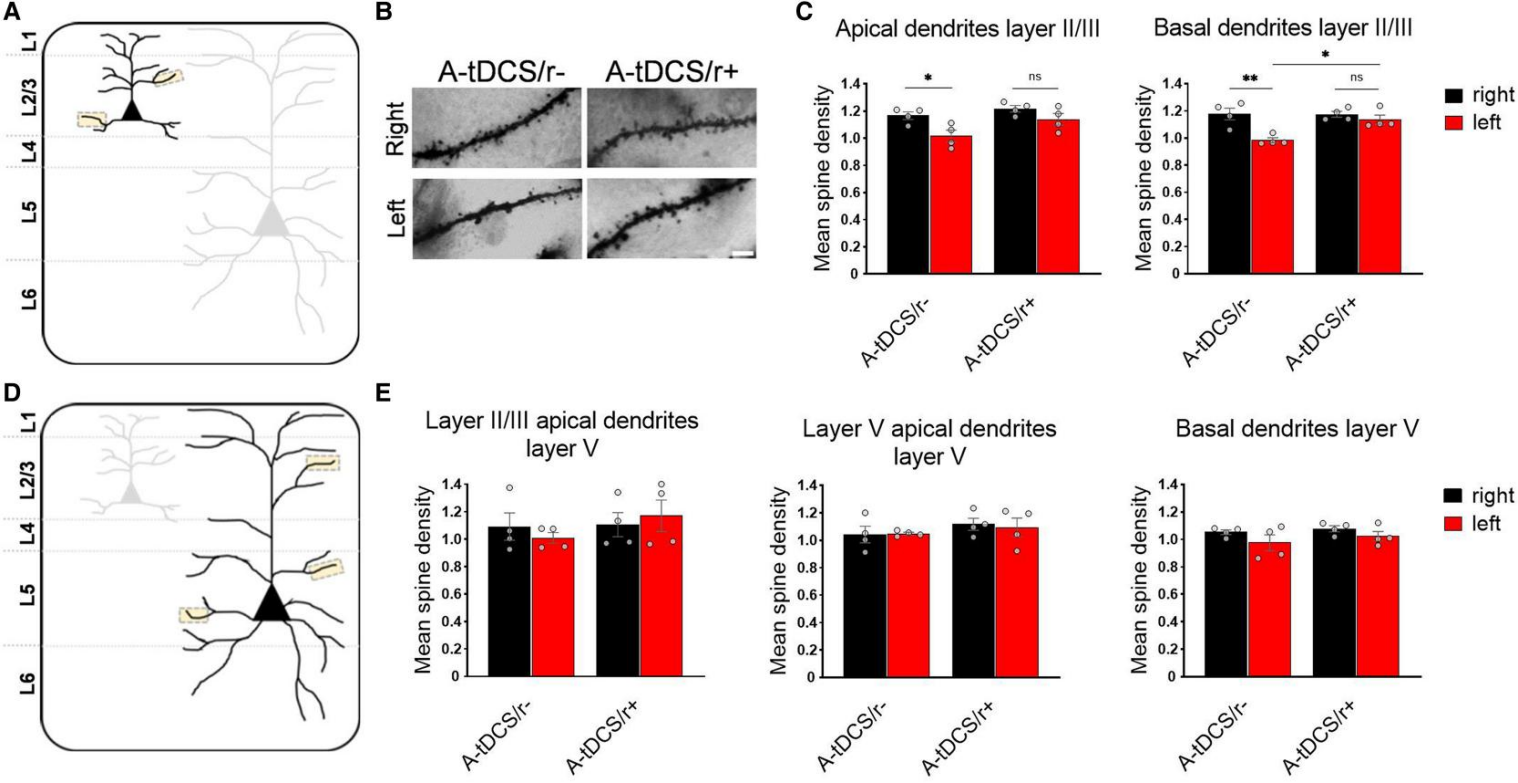
**A-tDCS coupled with walking induces distinct modulatory effects on the density of dendritic spines.** (**A**) Schematic illustrating the different regions included for spine quantification (yellow boxes) in layer II/III M1 pyramidal neurons. (**B**) Golgi-impregnated layer II/III M1 apical dendrites representative images. Images were acquired at 100X oil-immersed brightfield microscope. Scale bar = 5 µm. (**C**) Mean spine density in the apical and basal dendrites of layer II/III pyramidal neurons was increased in both M1 if A-tDCS was administered while walking. Analysis with one-way ANOVA followed by Tukey’s *post hoc* correction for apical dendrites: *F*_3,12_ = 5.779, *P* = 0.011; for basal dendrites: (*F*_3,12_ = 8.659, *P* = 0.0025); *N* = 4/group. (**D**) Schematic representation of dendritic segments analyzed in layer V. (**E**) Bar graphs representing the mean spine density in layer II/III apical (left), layer V apical (middle) and basal (right) dendrites of layer V M1 pyramidal neurons. Analysis with one-way ANOVA followed by Tukey’s *post hoc* correction; *N* = 4/group. Grey dots indicate individual values. Data are expressed as mean ± SEM. * *P* < 0.05; ** *P* < 0.01.

Structural plasticity, such as the dynamic changes in spine number, is associated with experience-dependent changes in neural circuits. Dendritic spines represent the post-synaptic partner of most excitatory input received by M1 neurons through interhemispheric connections, and dendritic spine density is known to play an important role in the formation and modulation of neural circuit functionality.^13^ In the sham groups, physical activity did not increase M1 mean spine density (Supplementary Fig. 3), in line with similar data in which mice were subject to slow constant-speed rotarod activity.^12^ On the contrary, (Fig. 4A–C) the application of A-tDCS increased the mean spine density in layer II/III apical (*P* = 0.04) and basal dendrites (*P* = 0.004), similarly to what was already observed after 3 sessions of A-tDCS in the awake mouse in layer II/III apical and basal dendrites by Barbati *et al*.^9^ Despite the absence of an independent effect of walking on spine density, the synergistic combination of walking and A-tDCS produced a marked increase in spine count throughout M1, encompassing both basal and apical dendritic compartments. Moreover, in basal dendrites, the A-tDCS/r+ unstimulated M1 showed higher spine density with respect to the A-tDCS/r- unstimulated cortex (*P* = 0.02; Fig. 4C). These data combined point towards A-tDCS acting synergistically with heightened M1 excitability, leading to an amplified effect. Analysis of layer V revealed no significant changes in activity due to either stimulation or walking (Fig. 4D, E), mirroring findings in anesthetized animals.^8^

**Figure 5:**
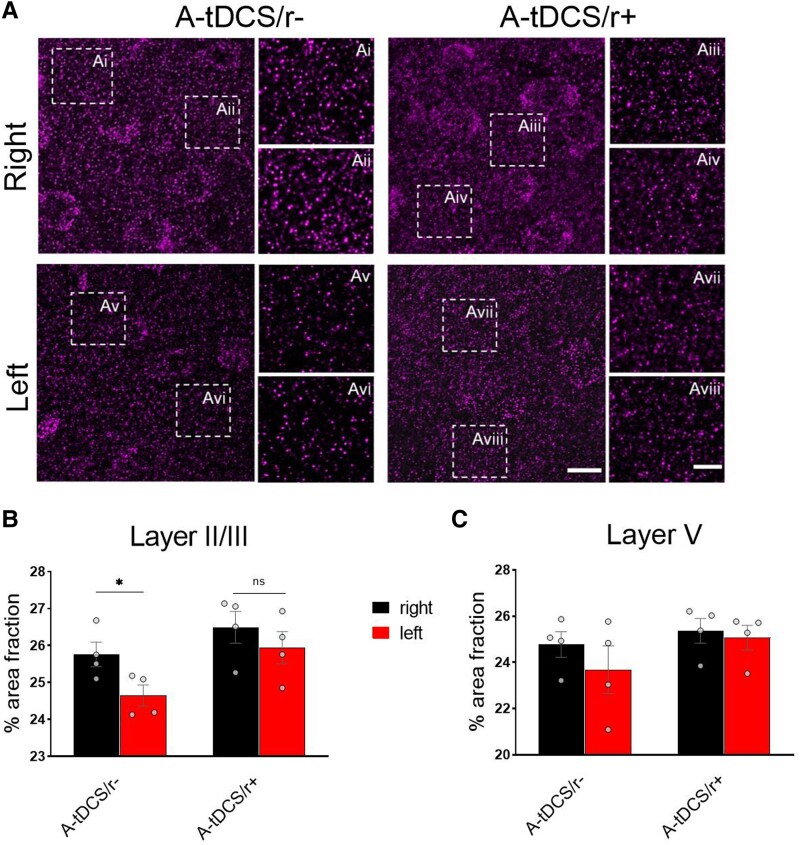
**The percentage of PSD95+ area fraction is increased in superficial layers in A-tDCS walking mice.** (**A**) Representative confocal images of right and left M1 in layer II/III, stained with anti-PSD95 antibody in A-tDCS/r− and A-tDCS/r+ conditions (63X magnification). Scale bar = 10 µm. Dotted squares represent the ROIs zoomed on the right. Scale bar = 2 µm. (**B, C**) Bar graphs show the % of area fraction of right and left layer II/III (**B**) and of layer V (**C**) M1 in A-tDCS/r− and A-tDCS/r+ conditions. Grey dots indicate individual values. *N* = 4/group. Data are expressed as mean ± SEM. Analysis with unpaired-*t* test. * *P* < 0.05.

The post-synaptic scaffolding protein 95 (PSD-95) exerts a key role in stabilising excitatory synapses and spines.^29^ To measure to what extent the increased spine density is related to synaptic potentiation when A-tDCS is applied during different brain states, in A-tDCS/r− and A-tDCS/r+, we measured the M1 percentage of PSD-95+ fraction area (Fig. 5A). In both conditions, PSD-95 signal was increased in the right M1 in layer II/III, with a significant effect in the A-tDCS/r− unstimulated side (i.e. left; *P* = 0.04), while A-tDCS/r + showed similar levels of PSD-95 expression (Fig. 5B). Analysis of PSD-95+ area in layer V, consistently with dendritic spine data, revealed no significant differences between groups (Fig. 5C).

## Discussion

While the crucial influence of brain activity during non-invasive stimulation has recently gained recognition, its underlying neuronal mechanisms and the full extent of its potential remain largely unexplored.^1,2,30-32^ For the first time, our research reveals that pairing monolateral M1 anodal tDCS with low-intensity physical activity promotes improvements in structural and functional plasticity across brain hemispheres in the behaving mouse model, under physiological conditions. This work extends our and others’ previous work, reporting the direct and indirect effects of A-tDCS on motor cortex plasticity when combined with its activation.^6-8^ Moreover, our results fit the theory that neuronal networks activated by a task (i.e. a motor task, in our case) are more sensitive to tDCS modulation,^33,34^ relative to the sole application of tDCS.

Activity-dependent plasticity is based on synaptic connection modulation within a network, contributing to changes in the connectivity patterns, thus enhancing pre-existing functional circuits or promoting the formation of new ones.^35^ In mice, the process of acquiring new motor skills is accompanied by modulations in the structure and function of synaptic spines, leading to an increased synaptic efficiency and strengthening of the horizontal connections within the motor cortex.^36,37^ Our results demonstrate that M1 activation elicited by A-tDCS alone (i.e. A-tDCS/r−) is accompanied by a rise in spine density and PSD-95 expression, predominantly in layer II/III, aligning with the observations of Barbati *et al*.^9^ The V layer, though being affected by the stimulation (i.e. increased levels of cFos expression versus sham), results in non-significant modulation in the number of dendritic spines in both basal and apical branching, as also for PSD-95 expression, which is known to improve synaptic stability and communication.^38^ This different pattern of activation may be attributed to two main aspects. First, deep layers are less affected by the stimulation, due to the well-known decrease in the electrical field strength beneath the electrode with distance.^39,40^ Second, mouse M1 layer II/III and layer V neurons demonstrate distinct connectivity, functional roles, and electrophysiological characteristics. Layer II/III neurons are essential for signal integration and the early stages of motor pattern learning, while layer V neurons play a more dominant role in controlling established motor behaviours.^41^ Based on our two-day stimulation protocol, it is plausible that layer II/III exhibits a higher level of activation compared with layer V, thus making it more responsive to tDCS. Similar to our findings, Gellner *et al*.^8^ observed no significant elevation in spine density 24 h following a 20-min anodal stimulation protocol in the apical tuft of layer V neurons. However, this finding was based on a single stimulation under ketamine anesthesia, which blocks NMDA receptors essential for long-lasting tDCS effects.^42^

Commonly, the rotarod task is adopted to assess rodents’ motor function, mainly endurance, and coordination, or to induce motor learning.^10^ Rotarod activity depends on the coordinated movement of both forelimbs and hindlimbs, thus requiring both right and left M1 activation. A low-intensity, continuous walking protocol was chosen to represent the physical activity component of this study. This choice was primarily to circumvent potential confounding factors, as slow rotarod exercise has shown limited or no impact on dendritic spine density,^12,43^ thus avoiding underestimating tDCS effects. Additionally, while intense physical exertion can lead to fatigue and decreased cortical activation, low or moderate exercise is linked to increased cortical excitability, at least in humans preconditioned with tDCS.^44^ Thus, when physical activity was coupled with A-tDCS (i.e. A-tDCS/r+), we observed a synergistic effect on the entire motor cortex activation, spine density, and intercortical M1-M1 synchronisation. The number of activated cells in the directly stimulated and non-stimulated hemisphere was increased with respect to the A-tDCS/r− group. In particular, the non-stimulated M1 had significantly higher activation in both layers II/III and V, revealing a robust indirect effect of tDCS in behaving mice. Of note, unilateral A-tDCS applied over the rat auditory cortex after the exposition of an intense tone resulted in morphological changes in both the stimulated and the unstimulated cortices,^45^ suggesting an inter-cortical stimulation effect also in sensory areas. As expected, the sham conditions resulted in lower cell activation, with a mild increased M1 activation of animals performing the rotarod. In A-tDCS/r+ mice, an increased spine density was found in layer II/III, while either layer V apical and basal dendrites did not show any difference with respect to the A-tDCS/r− group. As discussed above, this may be attributed to the fact that layer II/III and layer V pyramidal neurons are the basis of different aspects linked to motor learning, with layer II/III neurons being more involved in the early phases of motor learning.^11,46-48^ Consistent with previous findings, A-tDCS upregulated PSD-95 expression in layer II/III of the stimulated hemisphere but had no effect on layer V. However, when combined with physical activity, PSD-95 levels significantly increased in both hemispheres and across cortical layers. The synaptic scaffolding protein PSD-95 participates in experience-driven synaptic strengthening directly,^29^ thus influencing brain network remodelling. Importantly, PSD-95 plays a critical role in the trafficking and localisation of AMPA and NMDA glutamate receptors,^49^ both essential for tDCS after effects.^50,51^ Since motor cortices are reciprocally anatomically connected,^27^ we sought to elucidate the functional implications of these structural modifications by quantifying M1-M1 synchronous activity. LFP analysis revealed that boosting tDCS with motor activity results in a pronounced increase in low-theta coherence, a neural oscillation pattern linked to rodent locomotion,^28,52^ within the motor cortex. Given that high coherence is interpreted as evidence for functional coupling between two regions, an increased cortico-cortical synchronisation suggests a higher M1-M1 crosstalk, reflecting a more precise pattern of activation and less unnecessary interference.^53^ In mice, the motor cortices coordinate sequential bimanual movements through direct axonal projections to the contralateral motor cortex.^26^ However, the functional relevance of this modulation in the interhemispheric coherence still has to be fully clarified. Research in healthy individuals has shown that alterations in brainwave coherence correlate with behavioural outcomes.^54^ Additionally, post-stroke studies have demonstrated that interhemispheric desynchronisation is a valuable indicator of upper limb motor function recovery and responsiveness to rehabilitation.^55^

The stimulation protocol we used falls within the safety range suggested by anodal stimulation in rats,^56^ and is widely adopted in mice.^6,19,57^ Concerning human studies, we applied a current density of 5.5 mA/cm^2^ for 10 min, which is much higher than clinical studies, where the standard is in the range 0.029–0.08 mA/cm^2^.^58^ In our previous work on A-tDCS modulation of M1 in mice, we found that a 10’ stimulation results in an effective increase of the motor pathway excitability.^6^ The protocol, which involves repeated sessions, was selected based on previous literature,^59,60^ in which tDCS aftereffects can be enhanced by repeated stimulations without inducing current-related tissue damage. In line with this, no histological alterations were observed in the region underneath the electrode. A second major difference with respect to human studies is the montage adopted. Here, we used a monopolar stimulation with two distant electrodes to avoid current shunting,^6^ whereas a bilateral montage is usually adopted in human subjects. Both differences should be considered when comparing translational approaches, as well as the marked differences in cortical architecture observed between humans and mice. Further studies are required to verify the duration of the effects we observed and to investigate the association between tDCS and more targeted physical exercises. Additionally, we selected only adult male mice to eliminate potential confounding effects of the estrous cycle on evoked structural plasticity and spine density found in female subjects, as well as to avoid the synaptic pruning typically observed in younger animals.^61,62^ Overall, our results suggest that the effects of tDCS, rather than a direct trigger of synaptic plasticity, strongly depend on the endogenous network dynamic activity, namely the ‘brain state’. Moreover, it is tempting to speculate that tDCS-induced excitability facilitates the coupling of firing among endogenously activated synapses, thereby specifically activating and strengthening the motor cortical region involved in the task. The observed outcomes provide a better understanding of the principles underlying neural network reorganisation at the structural and functional levels within the motor cortex under physiological conditions. Those are relevant for understanding learning and memory formation and for treating neurological disorders, such as stroke.

## Supplementary Material

fcaf167_Supplementary_Data

## Data Availability

The data that support the findings of this study are available from the corresponding author on request. The accompanying code is provided as Supplementary material.
