# Supplementary material for: Physical activity and anodal-transcranial direct current stimulation: a synergistic approach to boost motor cortex plasticity
Source: Brain Commun. 2025 May 6;7(3):fcaf167. doi: 10.1093/braincomms/fcaf167 (PMC12062577; doi:10.1093/braincomms/fcaf167)
Supplement: fcaf167_Supplementary_Data [file fcaf167_supplementary_data.pdf]

Supplementary Material

Physical activity and A-tDCS: a synergistic approach to boost motor cortex plasticity

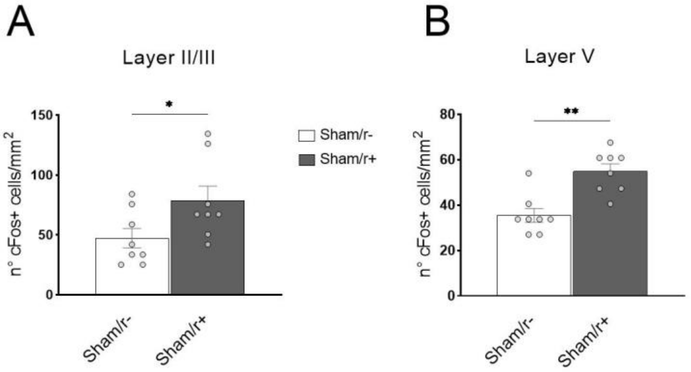

Supplementary Fig. 1. cFos+ cell count increases with moderate physical activity. The number of cFos+ cells in the two non-stimulated conditions in layer II/III (***A***) and layer V (***B***), when left and right hemisphere values are unified, show increased activity during walking. Gray dots indicate individual values. Analysis with unpaired t-test. n=8/group. Data are expressed as mean ± SEM. * $p<0.05$; ** $p<0.01$.

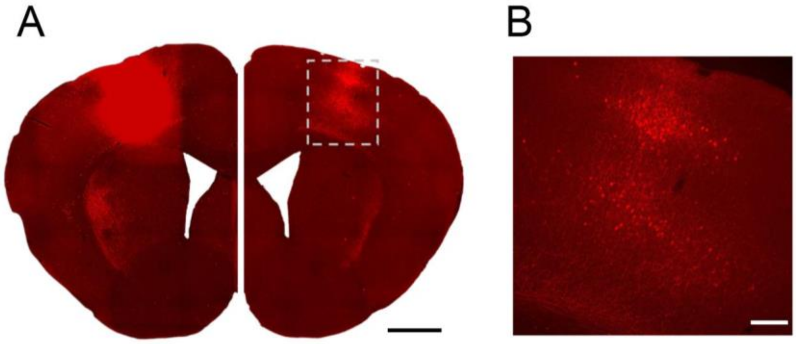

Supplementary Fig. 2. Cortico-cortical connections between M1. The panel shows the injection site (right M1) and the contralateral CTB-positive left M1 neurons (***A***), concentrated in layer II/III and layer V (***B***). Scale bar, 1 mm and 500 µm, respectively.

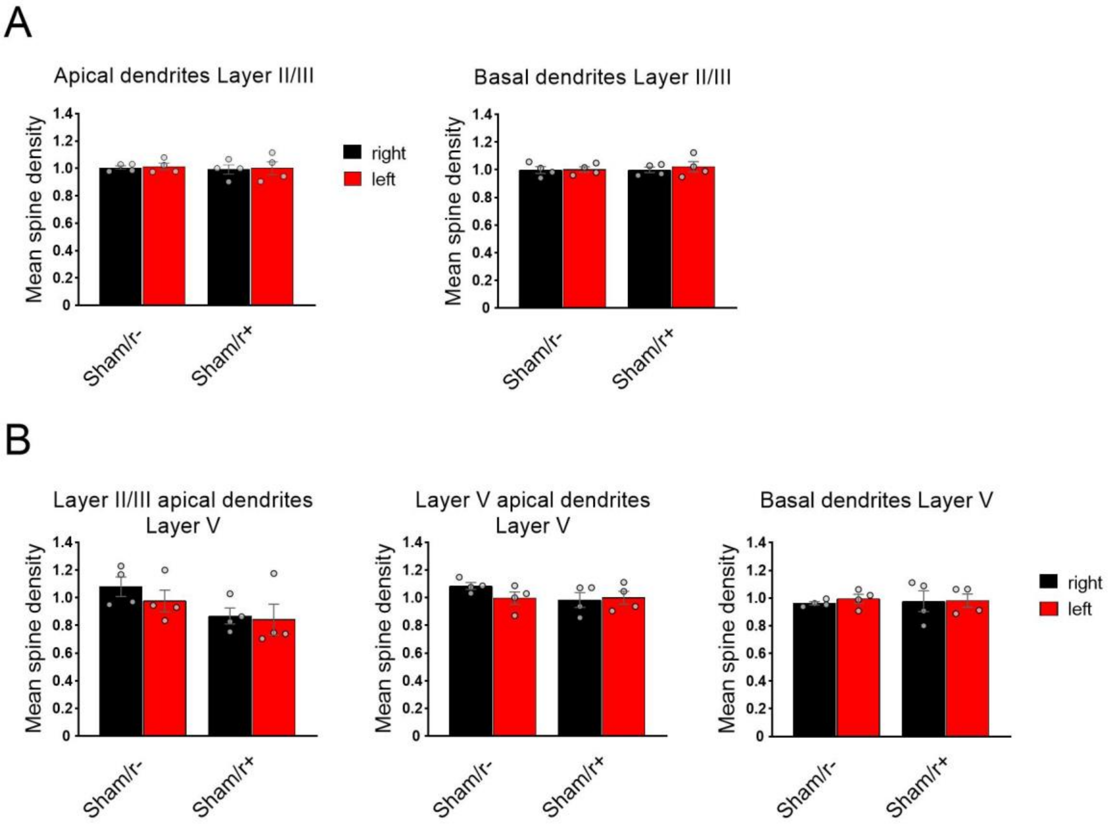

Supplementary Fig. 3. Two days of moderate walking on the rotarod did not modulate dendritic spine density. ***A.*** Bar graphs representing the mean spine density in the distal apical (left) and basal (right) dendrites of layer II/III pyramidal neurons, unchanged in the S/r- and S/r+ groups. ***B.*** Similarly, the spine density of layer V M1 pyramidal neurons remained unchanged in layer II/III apical (left), layer V apical (middle), and basal (right) dendrites. Analysis with One-way ANOVA followed by Tukey's Post hoc correction. Gray dots indicate individual values. n=4/group. Data are expressed as mean ± SEM.

LAYER II/III (n° cFos+ cells/mm^2)					
**Tukey's multiple comparisons test**	**Mean 1**	**Mean 2**	**Mean Difference**	**P Value**	**Summary**
Sham/r-:right vs. Sham/r-:left	48,32	46,22	2,101	>0,9999	ns
Sham/r-:right vs. Sham/r+:right	48,32	73,53	-25,21	>0,9999	ns
Sham/r-:right vs. Sham/r+:left	48,32	84,03	-35,71	>0,9999	ns
**Sham/r-:right vs. A-tDCS/r-:right**	**48,32**	**904,4**	-856,1	**<0,0001**	***
Sham/r-:right vs. A-tDCS/r-:left	48,32	307,8	-259,5	0,3025	ns
**Sham/r-:right vs. A-tDCS/r+ :right**	**48,32**	**1944**	-1896	**<0,0001**	***
**Sham/r-:right vs. A-tDCS/r+ :left**	**48,32**	**909,7**	-861,3	**<0,0001**	***
Sham/r-:left vs. Sham/r+:right	46,22	73,53	-27,31	>0,9999	ns
Sham/r-:left vs. Sham/r+:left	46,22	84,03	-37,82	>0,9999	ns
**Sham/r-:left vs. A-tDCS/r-:right**	**46,22**	**904,4**	-858,2	**<0,0001**	***
Sham/r-:left vs. A-tDCS/r-:left	46,22	307,8	-261,6	0,2935	ns
**Sham/r-:left vs. A-tDCS/r+ :right**	**46,22**	**1944**	-1898	**<0,0001**	***
**Sham/r-:left vs. A-tDCS/r+ :left**	**46,22**	**909,7**	-863,4	**<0,0001**	***
Sham/r+:right vs. Sham/r+:left	73,53	84,03	-10,5	>0,9999	ns
**Sham/r+:right vs. A-tDCS/r-:right**	**73,53**	**904,4**	-830,9	**<0,0001**	***
Sham/r+:right vs. A-tDCS/r-:left	73,53	307,8	-234,2	0,423	ns
**Sham/r+:right vs. A-tDCS/r+ :right**	**73,53**	**1944**	-1871	**<0,0001**	***
**Sham/r+:right vs. A-tDCS/r+ :left**	**73,53**	**909,7**	-836,1	**<0,0001**	***
**Sham/r+:left vs. A-tDCS/r-:right**	**84,03**	**904,4**	-820,4	**<0,0001**	***
Sham/r+:left vs. A-tDCS/r-:left	84,03	307,8	-223,7	0,479	ns
**Sham/r+:left vs. A-tDCS/r+ :right**	**84,03**	**1944**	-1860	**<0,0001**	***
**Sham/r+:left vs. A-tDCS/r+ :left**	**84,03**	**909,7**	-825,6	**<0,0001**	***
**A-tDCS/r-:right vs. A-tDCS/r-:left**	**904,4**	**307,8**	596,6	**0,0003**	**
**A-tDCS/r-:right vs. A-tDCS/r+ :right**	**904,4**	**1944**	-1040	**<0,0001**	***
A-tDCS/r-:right vs. A-tDCS/r+ :left	904,4	909,7	-5,252	>0,9999	ns
**A-tDCS/r-:left vs. A-tDCS/r+ :right**	**307,8**	**1944**	-1637	**<0,0001**	***
**A-tDCS/r-:left vs. A-tDCS/r+ :left**	**307,8**	**909,7**	-601,9	**0,0003**	**
**A-tDCS/r+ :right vs. A-tDCS/r+ :left**	**1944**	**909,7**	1035	**<0,0001**	***

Supplementary Table 1. Number of cFos$^+$ cells/mm^2 in M1 layer II/III

LAYER V (n° cFos+ cells/mm^2)					
Tukey's multiple comparisons test	Mean 1	Mean 2	Mean Difference	P Value	Summary
Sham/r-:right vs. Sham/r-:left	37,16	33,78	3,378	>0,9999	ns
Sham/r-:right vs. Sham/r+:right	37,16	55,74	-18,58	0,9593	ns
Sham/r-:right vs. Sham/r+:left	37,16	54,05	-16,89	0,9755	ns
**Sham/r-:right vs. A-tDCS/r-:right**	**37,16**	**157,1**	**-119,9**	**<0,0001**	*******
Sham/r-:right vs. A-tDCS/r-:left	37,16	77,7	-40,54	0,3279	ns
**Sham/r-:right vs. A-tDCS/r+ :right**	**37,16**	**314,2**	**-277**	**<0,0001**	*******
**Sham/r-:right vs. A-tDCS/r+ :left**	**37,16**	**158,8**	**-121,6**	**<0,0001**	*******
Sham/r-:left vs. Sham/r+:right	33,78	55,74	-21,96	0,9074	ns
Sham/r-:left vs. Sham/r+:left	33,78	54,05	-20,27	0,9368	ns
**Sham/r-:left vs. A-tDCS/r-:right**	**33,78**	**157,1**	**-123,3**	**<0,0001**	*******
Sham/r-:left vs. A-tDCS/r-:left	33,78	77,7	-43,92	0,2409	ns
**Sham/r-:left vs. A-tDCS/r+ :right**	**33,78**	**314,2**	**-280,4**	**<0,0001**	*******
**Sham/r-:left vs. A-tDCS/r+ :left**	**33,78**	**158,8**	**-125**	**<0,0001**	*******
Sham/r+:right vs. Sham/r+:left	55,74	54,05	1,689	>0,9999	ns
**Sham/r+:right vs. A-tDCS/r-:right**	**55,74**	**157,1**	**-101,4**	**0,0001**	******
Sham/r+:right vs. A-tDCS/r-:left	55,74	77,7	-21,96	0,9074	ns
**Sham/r+:right vs. A-tDCS/r+ :right**	**55,74**	**314,2**	**-258,4**	**<0,0001**	*******
**Sham/r+:right vs. A-tDCS/r+ :left**	**55,74**	**158,8**	**-103**	**0,0001**	******
**Sham/r+:left vs. A-tDCS/r-:right**	**54,05**	**157,1**	**-103**	**0,0001**	******
Sham/r+:left vs. A-tDCS/r-:left	54,05	77,7	-23,65	0,8709	ns
**Sham/r+:left vs. A-tDCS/r+ :right**	**54,05**	**314,2**	**-260,1**	**<0,0001**	*******
**Sham/r+:left vs. A-tDCS/r+ :left**	**54,05**	**158,8**	**-104,7**	**<0,0001**	*******
**A-tDCS/r-:right vs. A-tDCS/r-:left**	**157,1**	**77,7**	**79,39**	**0,003**	*****
**A-tDCS/r-:right vs. A-tDCS/r+ :right**	**157,1**	**314,2**	**-157,1**	**<0,0001**	*******
A-tDCS/r-:right vs. A-tDCS/r+ :left	157,1	158,8	-1,689	>0,9999	ns
**A-tDCS/r-:left vs. A-tDCS/r+ :right**	**77,7**	**314,2**	**-236,5**	**<0,0001**	*******
**A-tDCS/r-:left vs. A-tDCS/r+ :left**	**77,7**	**158,8**	**-81,08**	**0,0023**	*****
**A-tDCS/r+ :right vs. A-tDCS/r+ :left**	**314,2**	**158,8**	**155,4**	**<0,0001**	*******

Supplementary Table 2. Number of cFos$^+$ cells/mm^2 in M1 layer V
